# eHealth in geriatric rehabilitation: an international consensus study

**DOI:** 10.1007/s41999-025-01170-7

**Published:** 2025-03-21

**Authors:** Jules J. M. Kraaijkamp, Anke Persoon, Niels H. Chavannes, Wilco P. Achterberg, Mohamed-Amine Choukou, Frances Dockery, Hyub Kim, Laura M. Pérez, José E. Pompeu, Eva Topinkova, Mark A. Vassallo, Andrea B. Maier, Clemens Becker, Yoshiaki Amagasa, Marije S. Holstege, Jolanda van Haastregt, Eléonore F. van Dam van Isselt

**Affiliations:** 1 Department of Public Health and Primary Care, Leiden University Medical Center, Leiden, The Netherlands; 2 University Network for the Care Sector Zuid-Holland, Leiden University Medical Center, Leiden, The Netherlands; 3 Department of Primary and Community Care, Radboud University Medical Center, Nijmegen, The Netherlands; 4 National eHealth Living Lab, Leiden, The Netherlands; 5 Department of Occupational Therapy, College of Rehabilitation Sciences, Rady Faculty of Health Sciences, University of Manitoba, Winnipeg, Canada; 6 Centre on Aging, University of Manitoba, Winnipeg, Canada; 7 Beaumont Hospital & Royal College of Surgeons in Ireland, Dublin, Ireland; 8 Department of Occupational Therapy, Far East University, Eumseong, Republic of Korea; 9 Research on Aging, Frailty and Care Transitions in Barcelona (REFiT-BCN), Parc Sanitari Pere Virgili and Vall d’Hebron Institute (VHIR), Barcelona, Spain; 10 Department of Physiotherapy, Speech Therapy and Occupational Therapy, School of Medicine, University of São Paulo, São Paulo, Brazil; 11 Department of Geriatrics and Internal Medicine, First Faculty of Medicine and General University Hospital, Prague, Czech Republic; 12 Faculty of Health and Social Sciences, University of South Bohemia, České Budějovice, Czech Republic; 13 Geriatric Medicine Society of Malta, Karin Grech Hospital, Pieta, Malta; 14 Healthy Longevity Program, Yong Loo Lin School of Medicine, National University of Singapore, Singapore, Singapore; 15 NUS Academy for Healthy Longevity, National University of Singapore, Singapore, Singapore; 16 Department of Human Movement Sciences, @AgeAmsterdam, Faculty of Behavioural and Movement Sciences, Vrije Universiteit Amsterdam, Amsterdam Movement Sciences, Amsterdam, The Netherlands; 17 Department of Clinical Gerontology, Robert-Bosch-Krankenhaus, Stuttgart, Germany; 18 Unit of Digital Geriatric Medicine, Geriatric Centre, University Clinic, Heidelberg, Germany; 19 Nursing home, Mukoudai, Tokyo, Japan; 20 Department of Research, Treatment and Advice Center, Omring, Hoorn, The Netherlands; 21 Inholland University of Applied Sciences, Research Group Geriatric Rehabilitation, Centre of Expertise Prevention in Health and Social Care, Faculty of Health, Sports and Social Work, Amsterdam, The Netherlands; 22 Department of Health Services Research, Faculty of Health Medicine and Life Sciences, CAPHRI Care and Public Health Research Institute, Maastricht University, Maastricht, The Netherlands; 23 Living Lab in Ageing and Long Term Care, Maastricht University, Maastricht, The Netherlands

**Keywords:** Geriatric rehabilitation, eHealth, Implementation, Consensus, Delphi

## Abstract

**Purpose:**

Current evidence on the use of eHealth in geriatric rehabilitation is limited. This aim of this study was to achieve international consensus on three key eHealth-related topics in geriatric rehabilitation: the use, domains, and scientific evaluation of eHealth. Additionally, we developed a model that provides insight into the use of eHealth in geriatric rehabilitation.

**Methods:**

An international, two-round Delphi study was conducted. Two models served as a framework for the initial statement draft, with a total of 28 statements based on our systematic review results, an international survey, and expert opinion. Eligible healthcare professionals working in geriatric rehabilitation facilities were recruited across 10 countries.

**Results:**

Eighty healthcare professionals participated in round one and 47 in round two. In the first round, consensus was obtained for 20 of the 28 statements (71%). Prior to round two, four statements were revised, two statements were combined, and one statement was removed. In round two, consensus was obtained on six statements, bringing the total to 26: three related to the use of eHealth, five to the domains of eHealth, and 18 related to the scientific evaluation of eHealth.

**Conclusion:**

International consensus has been reached on the use, domains, and scientific evaluation of eHealth in geriatric rehabilitation. This first step in generating reliable knowledge and understandable information will help promote a consistent approach to the development, implementation, and scientific evaluation of eHealth in geriatric rehabilitation.

## Key summary points

**Aim:**

To achieve international consensus on three key eHealth-related topics in geriatric rehabilitation: the use, domains and scientific evaluation of eHealth.

**Findings:**

Eighty healthcare professionals participated in an two-round Delphi study. consensus was obtained on 26 statements: three related to the use of eHealth, five to the domains of eHealth and 18 related to the scientific evaluation of eHealth.

**Message:**

International consensus on eHealth in geriatric rehabilitation is achievable and essential for promoting a more consistent approach to the development, implementation, scientific and safety evaluation of eHealth.

## Introduction

Against a background of an aging population, demand for geriatric rehabilitation is expected to increase substantially and will require new strategies to maintain accessible and affordable service provision. eHealth has the potential to both improve quality and preserve accessibility of geriatric rehabilitation [1–4], but the integration of eHealth in geriatric rehabilitation remains challenging [5–11].

Over the last decade, a number of definitions of eHealth have been proposed but perhaps the most commonly used and easy-to-understand states: *“The use of digital information and communication to support and/or improve health and health care*” [12]. eHealth can be applied to various domains during geriatric rehabilitation. For instance, within the monitoring domain wearable sensors can reliably and objectively assess physical activity and sedentary behavior during rehabilitation. However, as the definition of eHealth is broad and therefore open to multiple interpretations depending on setting and context, healthcare professionals and patients in geriatric rehabilitation may have different ideas when they think or talk about use of eHealth. This may in turn negatively affect the acceptance and implementation of eHealth in geriatric rehabilitation [8, 9, 13].

In addition, the substantial increase in the variety and number of eHealth interventions has led to a rapid evolution of the eHealth landscape, resulting in scientific evidence on eHealth interventions that is diverse and often lacks usability outcomes [1, 13–15]. This lack of usability outcomes is particularly concerning given that age-related barriers may hinder the use of eHealth [16, 17]. Another concern is that patients and healthcare professionals will have difficulty identifying eHealth interventions that are effective, safe, valid, and suitable to their specific needs and context [8, 18].

International consensus on the description and evaluation of eHealth will promote a more consistent approach globally to the development, implementation, and scientific evaluation of eHealth in geriatric rehabilitation. In addition, a visual model that provides insight into the use and domains of health could help effectively present eHealth information and in a format, accessible for patients and healthcare professionals in geriatric rehabilitation. Therefore, the aims of this study included (1) reaching international consensus on three key topics related to eHealth in geriatric rehabilitation, namely, the use of eHealth, its domains, and scientific evaluation, and (2) creating a visual model that clearly explains the use and domains of eHealth in geriatric rehabilitation.

## Methods

### Design

An international online Delphi study [10, 19] was conducted between October 2022 and June 2023. Two models were developed to serve as a framework for the initial draft of statements, and several statements were prepared for each of the three topics (use, domains, and evaluation of eHealth). These statements were based on results from our international survey, expert opinions from researchers, and findings from our systematic review on eHealth in geriatric rehabilitation. Our review concluded that eHealth has the potential to improve rehabilitation outcomes, but the lack of usability outcomes might hinder its implementation [1, 18].

### Study population and setting

**Fig. 1:**
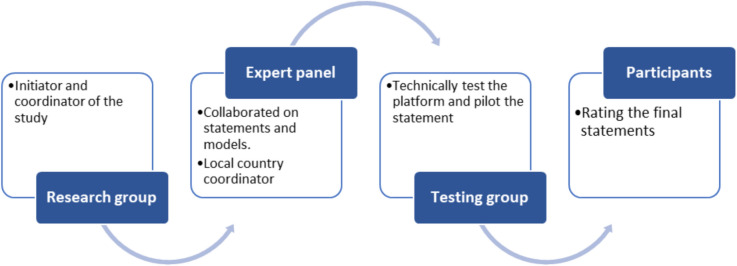
Participating groups and their different roles

Four different groups with distinct roles participated in the study, including a research group, an expert panel, a testing group, and participants: 1) The Research group initiated and coordinated the study. 2) The Expert panel, consisting of ten experts in eHealth in geriatric rehabilitation, collaborated on the conceptualization of both models, the composition and formulation of the statements, and discussed the results and the adjusted statements for subsequent rounds. Almost all initial experts were members of the European Geriatric Medical Society’s “Special Interest Group for Geriatric Rehabilitation” and were recruited through this network [20, 21]. Subsequently, expert members from outside Europe were invited to join the expert panel. Each member of the expert panel acted as a local country coordinator and was responsible for distributing the statements to participants in their country. 3) The Testing group compromised a group of five professionals who were approached by the research group to carry out technical testing of the platform and pilot the statements. 4) The participants included healthcare professionals with experience of eHealth in geriatric rehabilitation who were (i) working in a geriatric rehabilitation setting, (ii) aged 18 years old and over, (iii) understood English, and (iv) had at least three months of experience working in a geriatric rehabilitation setting. A local expert panel member approached participants with a request to rate the statements. The participating groups and their different roles are illustrated in Fig. 1.

### Recruitment and consent

Eligible participants were recruited in geriatric rehabilitation services across 10 countries: Brazil, Canada, Czech Republic, Germany, Ireland, Japan, Malta, The Netherlands, South Korea, and Spain. The distribution of statements varied by country depending on the personal preferences and experiences of the local expert panel member. Invitations included a link to a web-based survey that hosted the online statements, study information outlining the purpose, expected duration (15 min), confidentiality of responses, and contact details of the principal investigator. Participants did not receive any form of compensation. To boost response rates, a reminder was sent to participants in each country two weeks after the initial invitation. The online survey was hosted by Castor Electronic Data Capture (Castor EDC; Castor, Amsterdam, The Netherlands).

### Development of models

Two models were developed based on the findings of our systematic review and international survey. The first model concerning the use of eHealth was based on the *“Healthcare value cycle” *[11], while the second model, focusing on the evaluation of eHealth, was based on the “eHealth Evaluation Cycle” [22]. Following an initial meeting, the expert panel members gave their opinions and feedback on the models. The research group then fine-tuned both models based on the feedback (see appendix, Figs 4 and 5). After the last Delphi round a final visual model (based on prior models, results of the Delphi rounds and feedback from the expert panel) was created to provide insight into the use and domains of eHealth in geriatric rehabilitation.

### Delphi rounds

#### Preparation of statements

**Fig. 2:**
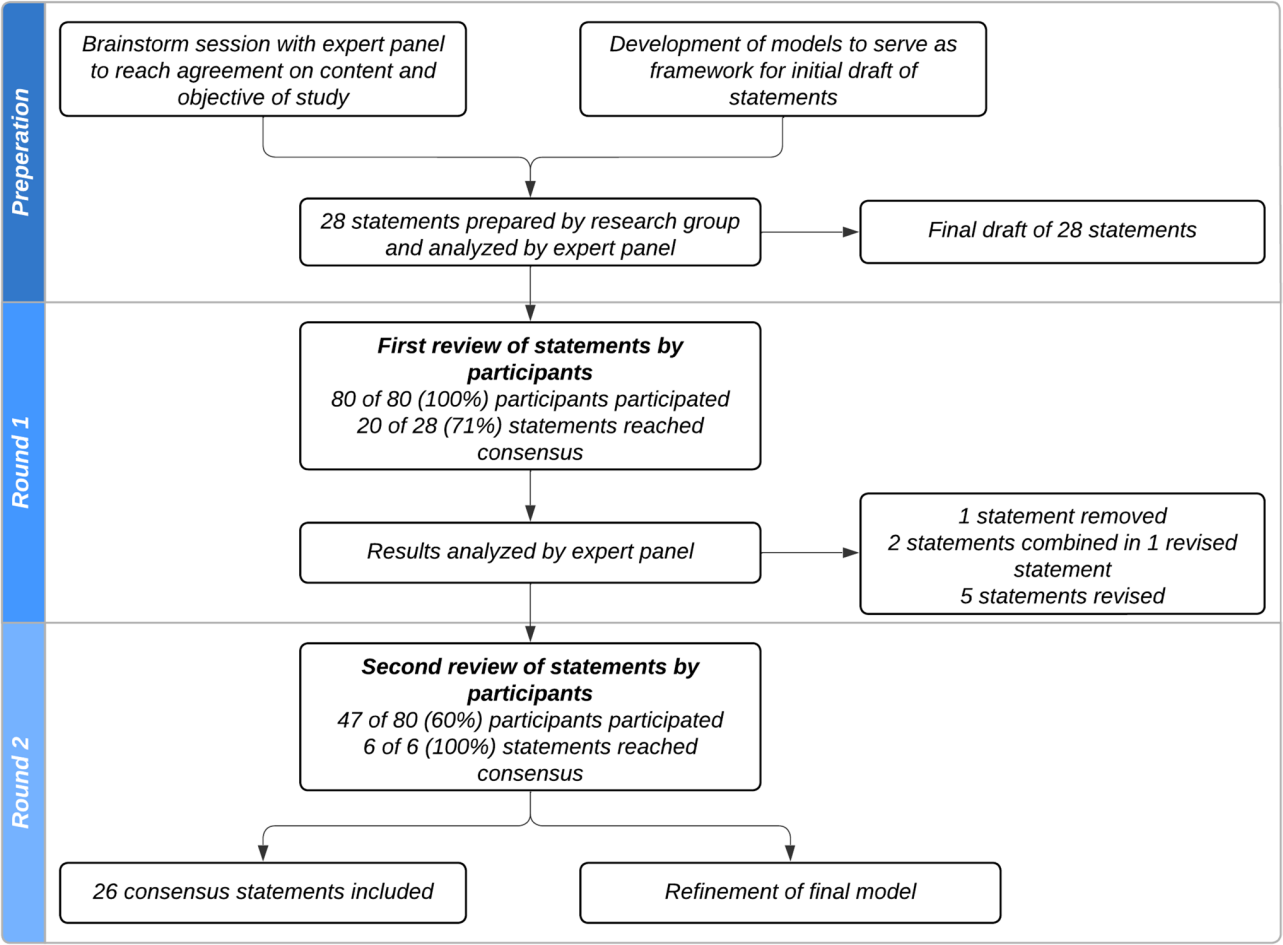
A flowchart illustrating the three phases of content preparation and consensus building

A flowchart presenting the different Delphi rounds is illustrated in Fig. 2. Prior to the first round of the study, members of the expert panel took part in an online semi-structured, open-ended brainstorming session primarily focusing on reaching agreement on the content and objectives of the consensus study. The research group began by presenting two models focused on the use and evaluation of eHealth in geriatric rehabilitation. The research group used the two models as a framework to draft an initial set of statements concerning the use, domains, and evaluation of eHealth in geriatric rehabilitation. The expert panel was consulted to gather feedback and the statements were adjusted based on their input. Finally, the testing group was consulted to technically assess the platform and pilot the statements.

#### Round 1

In the first round, participants were invited to rate each statement on a 5-point Likert scale ranging from 1 to 5, based on the level of agreement (1 = full disagreement to 5 = full agreement). Consensus on a statement was defined as 80% or more participants rating it as 4 or 5 (slight agreement, full agreement). Additionally, participants had the opportunity to clarify their answers by adding comments. The survey included 5 statements related to the use of eHealth, 5 statements about the domains of eHealth and 18 statements related to the scientific evaluation of eHealth in geriatric rehabilitation. Sociodemographic and professional characteristics of participants (sex, age, profession, working years, and country of origin) were collected. The research group analyzed the data from round one and when consensus was reached on a statement it was removed from consideration in the second round. Free-text comments from participants were reviewed by the research group and used to revise or remove statements that did not achieve consensus. This analysis was then presented and discussed with the expert panel.

#### Round 2

All members of the expert panel were requested to invite participants from the previous round. The participants' countries of origin were collected. At this stage, results from the previous round were presented to the participants. Each participant was asked to consider the mean score from the previous round and the adjustments made to the statements before re-rating them a second time. In the second and final round the survey included 2 statements related to the use of eHealth and 4 statements related to the evaluation of eHealth in geriatric rehabilitation.

### Statistical analyses

Descriptive statistics and frequency distributions were used to describe outcomes for the various statements. Surveys that were less than 90% complete were excluded from the final data analysis. Data were analyzed with SPSS version 25.0.

### Ethical considerations

Study approval was required and obtained in Ireland and the Netherlands, where required by local Medical Ethics Committee regulations. In other participating countries, ethical approval was not required. All participants signed the informed e-consent by clicking a dedicated button available in the invitation link, and by doing so they stated that they were aware that participation was voluntary.

## Results

A total of 80 participants took part in round one and 47 in round two. Participant characteristics are presented in Table 1. The median age of participants was 41 years (IQR: 29–51), the median number of years of work experience within geriatric rehabilitation was 10 (IQR 5–20), and most participants were from Europe (61%).

**Table 1:** Sociodemographic and professional characteristics of participants round 1 (*n* = 80)

	*n* (%)
Sex	
Female	67 (84)
Male	11 (14)
Prefer not to say	2 (2)
Age	
18–29	8 (10)
30–39	25 (31)
40–49	26 (33)
50–59	14 (17)
> 60	7 (9)
Profession	
Physiotherapist	30 (38)
Medical practitioner/ geriatrician	14 (18)
Occupational therapist	17 (21)
Other*	19 (23)
Working years	
1–5	28 (35)
6–15	29 (36)
16–25	12 (15)
> 25	11 (14)
Continent	
Europe (including the United Kingdom and Ireland)	49 (61)
North and South America	29 (36)
Asia	2 (3)

*Profession, other: researcher, dietician, manager, nurse, respiratory therapist, speech therapist

### Delphi round 1

The results of all Delphi rounds are presented in Tables 2, 3, and 4. In the first round consensus was obtained on 20 of the 28 statements (71%), including consensus on 1 of 5 (20%) statements related to the use of eHealth, 5 of 5 (100%) related to the domains of eHealth, and 14 of 18 (78%) statements related to the scientific evaluation of eHealth in geriatric rehabilitation.

**Table 2:** Statements and revised statements related to use of eHealth, results from rounds 1 and 2

Statements	Median (IQR)	Consensus reached* Y/N (%)
A more specific description of eHealth in GR, including the use, domains, and evaluation eHealth in GR, is needed to achieve a more consistent approach of eHealth in GR	5 (1)	Y (84)
eHealth in GR should primarily focus on		
(Patient-centered) rehabilitation goals	4 (1)	N (76)
The interaction between the patient / caregiver and the healthcare professional	4 (2)	N (72)
eHealth in GR should preferably be delivered as blended care (a combination of traditional face-to-face and online care (eHealth))	5 (1)	N (78)
Big data, artificial intelligence, and prediction models are important topics for the future use of eHealth in GR	4 (2)	N (57)
Revised statements from second round		
eHealth in geriatric rehabilitation should focus primarily on monitoring, training, and self-management, and secondarily on information and counseling	4 (1)	Y (83)
eHealth in geriatric rehabilitation should preferably be integrated into care pathways (blended care, hybrid care)	5 (1)	Y (94)

*Consensus: % of participants who rated a 4 or 5 (slightly agreement, full agreement)

**Table 3:** Statements related to the domains of eHealth, results from round 1 (n = 5)

Statements	Median (IQR)	Consensus reached* Y/N (%)
For eHealth in GR it is beneficial to focus on several specific domains:		
Monitoring	4 (1)	Y (86)
Training	4 (1)	Y (87)
Self-management	5 (1)	Y (85)
Information	4 (1)	Y (80)
Consultation	4 (1)	Y (81)

*Consensus: % of participants who rated a 4 or 5 (slightly agreement, full agreement)

**Table 4:** Statements and revised statements related to the evaluation of eHealth, results from rounds 1 and 2

Statements	Median (IQR)	Consensus reached* Y/N (%)
For the development and evaluation of eHealth in GR it is useful to use the “eHealth evaluation cycle.”	4 (1)	n (74)
Patients and professionals should be involved during each phase of the “eHealth evaluation cycle.”	5 (1)	Y (89)
The following outcome domains should be included when evaluating eHealth in GR:		
Usability (the extent to which a system, product, or service can be used by specified users to achieve specified goals with effectiveness, efficiency, and satisfaction in a specified context of use)	5 (0)	Y (95)
Digital health literacy	5 (1)	Y (85)
Experiences/satisfaction	5 (1)	Y (94)
Adverse outcomes	5 (1)	Y (87)
(Cost)-effectiveness	4 (1)	N (79)
Organization and local aspects (feasibility)	4 (1)	Y (90)
Technical aspects	4.5 (1)	Y (81)
Interoperability (a characteristic of a product or system to work with other products or systems)	4 (1)	Y (84)
Adherence/uptake	5 (1)	Y (91)
Outcome domains related to effectiveness should be structured using the following classification systems: The International Classification of Functioning, Disability and Health classification system (ICF)	4 (2)	N (65)
Outcome measures related to usability should include clear endpoints or reliable and validated questionnaires	5 (1)	Y (88)
Outcome measures related to usability should include one or more of the following age-related barriers:		
Cognition	5 (0)	Y (93)
Physical ability	5 (1)	Y (87)
Motivation	5 (1)	Y (85)
Perception	4 (1)	N (78)
Guidance and support (describe usability problems that occur when the eHealth intervention does not provide sufficient support and feedback for tasks that the user must perform and (potential) errors the user makes)	5 (1)	Y (81)
Revised statements from second round		
For the development and evaluation of eHealth in GR it is advised to use evidence-based evaluation frameworks such as the “eHealth evaluation cycle.”	5 (1)	Y (92)
It is advised that outcome domains related to effectiveness should be structured using a classification system such as the Classification of Functioning, Disability and Health (ICF) or the Post-acute care rehabilitation quality model (Jesus and Hoenig, 2015)	5 (1)	Y (96)
It is advisable to include (cost) effectiveness as an outcome domain in the evaluation of eHealth in GR	5 (1)	Y (94)
Depending on the type of eHealth intervention, outcome measures related to usability should include the following age-related barriers: perception	5 (1)	Y (85)

Consensus: % of participants who rated a 4 or 5 (slightly agreement, full agreement)

### Delphi round 2

Before the 2nd round, adjustments were discussed with the expert panel regarding the 8 statements on which no consensus was reached in round 1. Among the statements on eHealth use, one statement regarding the use of big data and artificial intelligence (AI) was removed because participants questioned its usefulness, ethical aspects, and alignment with the topic. Two other statements were combined into one revised statement. Additionally, four statements on the topic *evaluation of eHealth* were revised based on participants’ comments from the first round. Participants were invited to respond to the six revised statements and consensus was reached on all statements. The participants' countries of origin are presented in Table 5.

**Table 5:** Country of origin from participants in round 2 (*n* = 46)

	*n* (%)
Continent	
Europe (including the United Kingdom and Ireland)	26 (55)
North and South America	8 (17)
Asia	13 (28)

### Final model

**Fig. 3:**
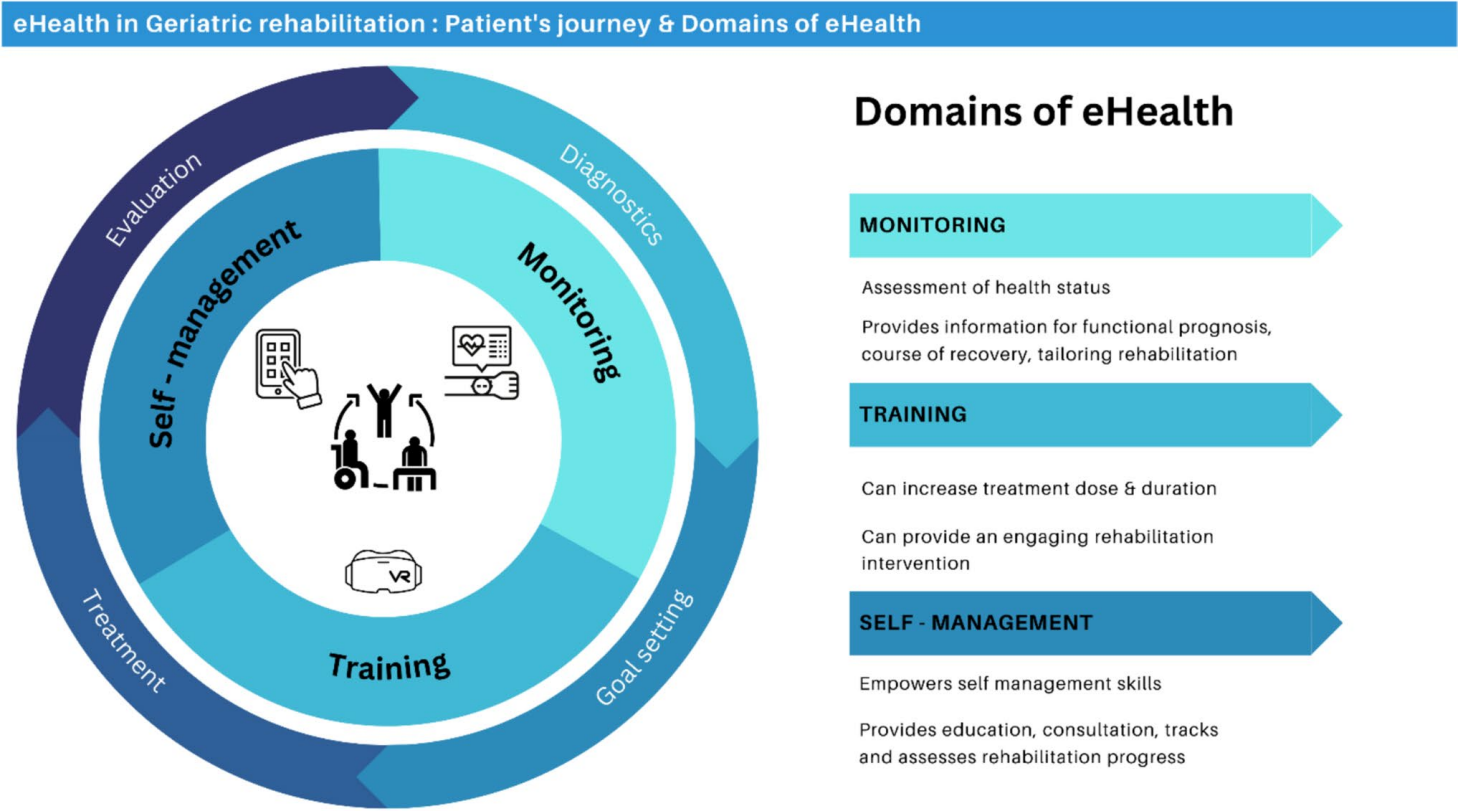
Final model patient’s journey and domains of eHealth

**Fig. 4:**
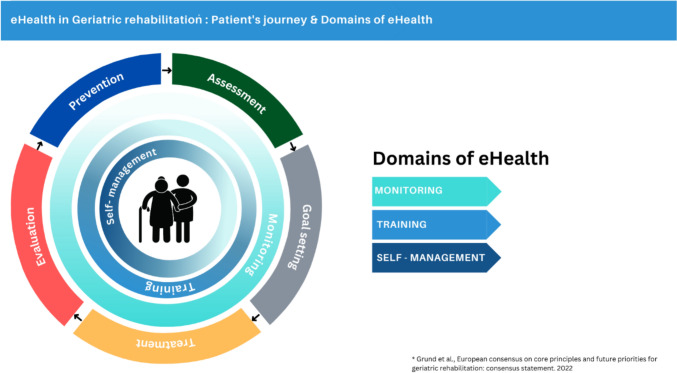
Original model: use of eHealth

**Fig. 5:**
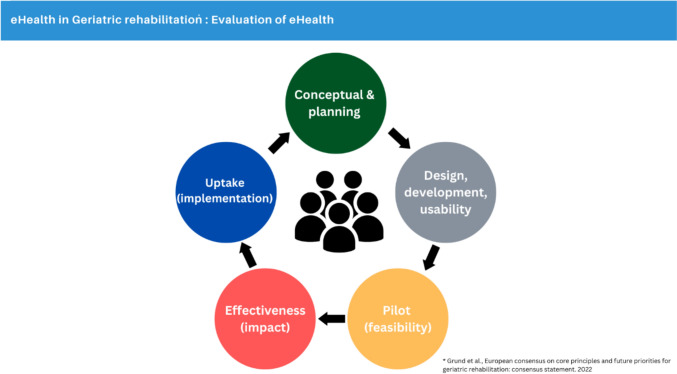
eHealth evaluation cycle

The final model is shown in Fig. 3. The model utilizes a patient journey to illustrate domains of eHealth that may be of added value at each phase of rehabilitation. A patient journey was incorporated into the final model in order to help patients and healthcare professionals in geriatric rehabilitation to better understand the use and timing of eHealth within the appropriate context. Since the information and consultation domains apply throughout the patient journey, both were incorporated into the other three domains (monitoring, training, and self-management).

### Free-text comments

The free-text comments gave important insights into the participants' rationale for the various statements. These points are reported under each topic below.

### Use of eHealth

Consensus was reached regarding the statement that “a more specific description of eHealth in geriatric rehabilitation is needed.” Several participants commented that this would promote a more consistent approach to explaining the concept of eHealth to patients and professionals in geriatric rehabilitation,* “I agree, especially concerning understanding the term eHealth and explaining it to patients and other professionals in GR.”* However, some participants commented that rather than a separate description specifically for geriatric rehabilitation a description for rehabilitation in general would be sufficient, *“I wonder if we need to differentiate eHealth for GR or more for rehabilitation in general.”*

There was no consensus on big data, machine learning and prediction models as important topics for the future use of eHealth in geriatric rehabilitation. Multiple participants expressed concerns regarding the ethical implications and privacy considerations related to use of artificial intelligence (AI*), “..I think great caution is needed due to substantial privacy concerns around big data and lax AI regulation. AI might have a role but it will require considerable forethought and caution, as well as consideration of what fully informed consent might look like in this situation.”*

### Domains of eHealth

In the context of eHealth for geriatric rehabilitation, there was consensus that it would be beneficial to focus on specific domains such as monitoring. Participants noted that use of monitoring would allow eHealth to measure outcomes, in turn helping establish quantifiable goals*. “I think monitoring helps provide data that can assist in determining baseline, progress and use to measure outcomes. It is the ‘measurable’ part when thinking about SMART goal setting.”* Similarly, there was agreement concerning a focus on training as a specific domain of eHealth. Participants commented that there is sufficient potential to justify integrating eHealth into rehabilitation treatment. *“To me, I love the idea of incorporating more of this type of technology into treatment. It keeps sessions interesting, can offer environments that are not always otherwise feasible and frees up personnel for other tasks (for example, a Rehab. Assistant or caregiver who may have to assist with the activity). Trainer and user familiarity with training devices may complicate their ability to support e-Health use.”*

### Evaluation of eHealth

Consensus was reached that Usability should be included as an outcome domain when evaluating eHealth in geriatric rehabilitation. Multiple respondents noted the importance of evaluating usability, especially in older adults with cognitive decline*. “Technology is not something that most of the geriatric population is familiar with; this of course will change in the future. We also need to take into consideration that people with cognitive impairment will have difficulty navigating technology.”*

There was consensus on the statement that outcome domains related to effectiveness should be structured using a classification system such as the Classification of Functioning, Disability and Health (ICF). Most participants found classification systems useful for structuring outcome domains, though some suggested that alternatives might be feasible. “The ICF is likely the one classification system that is ‘universally accepted’ in rehabilitation, but there may be others that are a better fit for eHealth initiatives.”

## Discussion

This study aimed to reach international consensus on three key topics related to eHealth in geriatric rehabilitation: the use of eHealth, its domains, and scientific evaluation. Based on a two-round Delphi method, 80 participants from 10 countries reached consensus on 26 eHealth statements: 3 on use, 5 on the domains, and 18 on the scientific evaluation of eHealth.

Our study also highlighted the need for a specific description of eHealth in geriatric rehabilitation. Over the years numerous definitions of “eHealth” have been proposed, for example, a 2005 systematic review found 51 unique but highly heterogeneous definitions of eHealth [23]. While the appearance of so many definitions shows the widespread recognition of eHealth, this diversity may result in fragmented understanding [24]. Our final model therefore aims to provide a specific description of the use and domains of eHealth in geriatric rehabilitation, providing clear and reliable eHealth information in a format equally accessible to patients and healthcare professionals in geriatric rehabilitation.

The only excluded statement concerned big data and AI, which was removed due to the many questions raised by participants regarding use and ethics. Big data and AI are undoubtedly promising, but as a fast-emerging technology there are serious concerns regarding safety, transparency, and accuracy [25–27], particularly as the complex and opaque relationships between input and output on which AI relies can yield errors that are difficult to foresee or prevent [24]. A recent systematic review identified 36 studies involving guidelines, consensus statements, and standards on the application of AI in health care [28], but specific guidelines and standards on the use of AI in geriatric rehabilitation are still needed. In addition, a recently proposed quality assessment framework provides guidance on the appropriate validation steps needed to ensure safe and reliable AI-based predictive models [29]. Such frameworks and guidelines can provide a starting point for the safe and responsible implementation of AI-based prediction models in geriatric rehabilitation.

An important achievement of the study was reaching consensus on all proposed statements related to the evaluation of eHealth in geriatric rehabilitation. Almost all participants agreed that specific outcome domains such as usability should be included when evaluating eHealth in geriatric rehabilitation. Additionally, agreement was reached on the importance of incorporating age-related outcomes such as cognition, physical ability, and motivation in these evaluations. The proposed age-related outcome domains were in line with the MOLD-US framework, which is an evidence-based framework of usability and age-related outcomes [16]. In current practice eHealth is often insufficiently tailored to age-related barriers, which hampers efficient use of eHealth and possibly results in non-adoption by older adults receiving geriatric rehabilitation [30]. Considering that current literature on the usability of eHealth in geriatric rehabilitation is very limited, and studies that do include usability outcomes show diverse results without clear outcome measures [1], reaching consensus on usability outcome domains is an important but challenging step toward more evidence-based practice of eHealth in geriatric rehabilitation.

A strength of this study was the involvement of professionals from several disciplines, making the study multidisciplinary in nature. Furthermore, the majority (65%) of participants had more than 10 years of working experience, enabling them to critically assess the proposed assessments. Nevertheless, several study limitations should be mentioned. While this study included 80 participants across a range of countries, the number of participants per country varied considerably and most participants were from countries within Europe. This inevitably leads to less reliable data. Due to the design of the digital Delphi rounds that assured participants' anonymity it was not possible to directly re-invite the exact same participants for the second Delphi round. The same members from the expert panel were asked to send the invitation to the same group of participants in their country, and since we only collected participants' countries of origin in the second Delphi round we could not verify whether the same participants re-evaluated the revised statements. To ensure participants in the second Delphi round could make informed decisions, the results from the first round were presented at the beginning of the second round. Furthermore, Boel et al. [31] demonstrated that including participants who missed a previous Delphi round does not affect the final outcome. Instead, it enhances the representation of diverse opinions and reduces the likelihood of false consensus. It is important to mention that since the survey was in English, participants in non-English-speaking countries might have been unable to participate or may have had difficulty articulating their responses clearly. This could have ultimately resulted in lower participation rates from those countries. Lastly, this study focused on the perspectives of healthcare professionals and did not consider the views of patients and caregivers. Future research on this topic are needed to incorporate their input on the current statements and gather additional feedback to refine the final model, ensuring it is both relevant and beneficial for them.

## Conclusion

Our primary conclusion is that it is possible to achieve broad international consensus on the use and evaluation of eHealth in geriatric rehabilitation. Achieving consensus on these topics is important since it will facilitate reliable, easily understandable information on eHealth in geriatric rehabilitation for patients, healthcare professionals, and researchers alike. Ultimately, this work may promote a more consistent approach to the development, implementation, and scientific and safety evaluation of eHealth on a global scale in this rapidly growing area of healthcare.

## Data Availability

The data presented in this study are available upon reasonable request from the corresponding author. Requests will be judged based on the originality of the research question and the feasibility of the analysis plan.

## Appendix

### Original models

See Figs. 4 and 5.

### Original online statements

#### Introduction

eHealth geriatric rehabilitation (GR) has the potential to improve outcomes that matter to patients, their caregivers, healthcare professionals, and society. However, eHealth is not yet sufficiently integrated in geriatric rehabilitation, mainly due to the implementation of eHealth being complex, time consuming, and because it often requires a change in workflow to successfully integrate eHealth in daily practice (1–5). Furthermore, the landscape of eHealth applications is changing rapidly, with a wide variety of eHealth interventions constantly being added, updated, or deleted. As a result, the scientific evidence on effectiveness, feasibility, and usability of these eHealth interventions often lags behind (6–8). In addition, eHealth interventions, especially mobile apps, have a broad quality range, are rarely subjected to rigorous scientific evaluation and often lack in privacy and security features (9). This makes it difficult for patients and healthcare professionals to comprehend which eHealth interventions are safe and suitable to fit their specific needs and contexts. Lastly, the current scientific evidence on eHealth in GR is diverse and often lacks specific age-related outcome domains such as usability, making it hard to compare outcomes and difficult to assess whether the examined eHealth intervention is usable for older adults (10).

The aim of this study is to develop a consensus on the use, domains, and evaluation of eHealth in GR with the potential to enable informed decision making, and facilitate uniformity in research through the use of evaluation frameworks and the standardization of outcome domains to ultimately promote the implementation and integration of eHealth in GR.

Several statements were prepared for each of the three topics. Each topic is accompanied by a brief introduction explaining the rationale for choosing this topic and what each topic covers. We would like to ask you to go through each statement carefully and rate the level of agreement (1 = full disagreement; 5 = full agreement). After each statement it is possible to comment on the content or formulation of the statement in the free text boxes.

**Topic 1: eHealth in GR**

In the last decade several definitions of eHealth have been proposed of which the most frequently used and easy-to-understand definition is “The use of digital information and communication to support and/or improve health and health care.”(11). However, this definition is very broad and therefore open to multiple interpretations depending on setting and context such as GR. In the absence of a more specific definition or description, patients and healthcare professionals in the GR may have a different understanding of what they mean when they think or talk about the use of eHealth and its goals, which in turn may negatively affect the acceptance of eHealth in the GR (4–6). Therefore, consensus on a more specific description and the main goals of eHealth in GR could help to achieve a more consistent approach in the development, implementation, and scientific evaluation of eHealth in GR. Furthermore, it could facilitate the provision of reliable and easily understood information on eHealth to patients and healthcare professionals in GR.

1.1 A more specific description of eHealth in GR, including the use, domains, and evaluation eHealth in GR, is needed to achieve a more consistent approach of eHealth in GR
1.2 eHealth in GR should primarily focus on:
  - (Patient-centered) Rehabilitation goals
  - The interaction between the patient / caregiver and the healthcare professional
  1.3 eHealth in GR should preferably be delivered as blended care (a combination of traditional face-to-face and online care (eHealth))
  1.4 Big data (a combination of structured, semi-structured, and unstructured data that can be used for information in machine learning) and prediction models are important topics for the future use of eHealth in GR

**Topic 2: Domains of eHealth**

A next step for a more specific description of eHealth is to specify the domains in which eHealth can be beneficial. For inspiration, we have developed a model that represents the patient's journey and covers different domains where eHealth can be beneficial. For each domain, we have added examples of different forms of eHealth that can be used. We developed this model based on findings from the literature, an international survey, and opinions of expert group involving healthcare professionals and researchers in the field of eHealth and GR. The model can be found here defining domains can help patients and healthcare professionals to better understand what eHealth in GR is truly about. Furthermore, defining domains also helps patients and healthcare professionals better understand which forms of eHealth are more suitable / appropriate in certain domains of GR.

2.1 For eHealth in GR, it is beneficial to focus on several specific domains (choose 1 or more):
  - Monitoring (see link to model for further explanation and examples)
  - Training (see link to model for further explanation and examples)
  - Self-management (see link to model for further explanation and examples)
  - Information
  - Consultation
  - Other, namely:
  - Other, namely:
  - Other, namely:

**Topic 3: Evaluation of eHealth**

eHealth solutions are considered complex interventions. Studying such interventions requires multiple evaluation approaches that can capture the complexity of successive phases of intervention, development, and implementation. Evaluating eHealth is critical before starting implementation and adoption of usable and effective eHealth programs. Additionally, for the evaluation of eHealth in GR, it may be useful to include specific outcome domains such as usability and digital health literacy, since these variables have a significant impact on successful implementation. Consensus on eHealth evaluation is needed to provide a clear overview of evaluation approaches that are suitable in GR, thereby facilitating the development and implementation of eHealth in GR.

3.1 For the development and evaluation of eHealth in GR it is useful to use the “eHealth evaluation cycle”
3.2 Patients and professionals should be involved during each phase of the “eHealth evaluation cycle”
3.3 The following outcome domains should be included when evaluating eHealth in GR (choose 1 or more):
  - The following outcome domains should be included when evaluating eHealth in GR (choose 1 or more):
  - Usability (The extent to which a system, product or service can be used by specified users to achieve specified goals with effectiveness, efficiency and satisfaction in a specified context of use)
  - Digital health literacy
  - Experiences/satisfaction
  - Adverse outcomes
  - (Cost)-effectiveness
  - Organization and local aspects (feasibility)
  - Technical aspects
  - Interoperability (a characteristic of a product or system to work with other products or systems)
  - Adherence/uptake
  - Other, namely:
  - Other, namely:
  - Other, namely:

3.4 Outcome domains related to **effectiveness** should be structured using the following classification systems:
  - The International Classification of Functioning, Disability and Health classification system (ICF), a classification of health and health-related domains, link
  - Other, namely:
  - Other, namely:
  - Other, namely:

3.5 Outcome measures related to **usability** should include clear endpoints or reliable and validated questionnaires
3.6 Outcome measures related to **usability** should include one or more of the following age-related barriers:
  - Cognition
  - Physical ability
  - Motivation
  - Perception
  - Guidance and support (describe usability problems that occur when the eHealth intervention does not provide sufficient support and feedback for tasks that the user must perform and (potential) errors the user makes)
  - Other, namely:
  - Other, namely:
  - Other, namely:
